# Influence of Sample Position on Strain Monitoring in Composite Materials Using Magnetic Microwires

**DOI:** 10.3390/s25164892

**Published:** 2025-08-08

**Authors:** Rafael Garcia-Etxabe, Maitane Mendinueta, Marta Camacho-Iglesias, Valentina Zhukova, Arcady Zhukov

**Affiliations:** 1 Department of Composites and Sustainable Functional Polymers, GAIKER, Basque Research and Technology Alliance (BRTA), Technology Park of Biscay, 48170 Zamudio, Spain; mendinueta@gaiker.es (M.M.); marta.camacho@gaiker.es (M.C.-I.); 2 Department of Electricity and Electronics, University of the Basque Country, UPV/EHU, 48940 Leioa, Spain; 3 Department of Polymers and Advanced Materials, University of the Basque Country, UPV/EHU, 20009 Donostia, Spain; 4 Department of Applied Physics, Escuela de Ingeniería de Gipuzkoa, EIG, University of the Basque Country, UPV/EHU, Plaza Europa 1, 20018 San Sebastian, Spain; arkadi.joukov@ehu.es; 5 EHU Quantum Center, University of the Basque Country, UPV/EHU, 20018 San Sebastian, Spain; 6 IKERBASQUE, Basque Foundation for Science, 48011 Bilbao, Spain

**Keywords:** composite material, magnetic microwire, structural health monitoring

## Abstract

Soft magnetic materials are highly suitable for use as sensors in the monitoring of materials, applications, and processes, with proven effectiveness across various industries. Their ability to be configured as microwires allows excellent integration within composite structures, making them particularly effective for structural health monitoring. Research in this area has enabled the analysis of both hysteresis loops and scattering parameters in transmission and reflection within the microwave frequency range, under conditions such as composite matrix polymerization or when subjecting specimens to different stress states. Consequently, a clear dependence of scattering parameters and impedance on applied stress in composites with magnetic microwire inclusions, which can be monitored, has been demonstrated. However, despite the repeatability of the phenomenon, modeling this behavior is challenging due to the dispersion of results caused by multiple factors and varying conditions that influence outcomes in a conventional environment. This study analyzes the influence of the relative sample position on these measurements and presents results obtained by modifying the position and orientation of microwires through rotation and flipping movements of the test specimen.

## 1. Introduction

Composite materials have demonstrated excellent structural performance and a high strength-to-weight ratio, making them ideal for transportation applications [1]. However, the use of composites in critical structures poses new challenges in terms of safety and maintenance. Detecting internal stresses, cracks, delaminations, and other structural defects during service life often incurs substantial costs due to the reliance on sophisticated sensors and characterization techniques required for periodic inspections and preventive maintenance. These efforts are essential to ensure system integrity is not compromised. As a result, recent years have seen an intensification in research into embedded sensing technologies capable of offering real-time structural monitoring in a non-invasive and wireless manner [2].

Previously described non-destructive testing methods using embedded sensors have a number of disadvantages, including changes in local mechanical properties and deterioration of sensing ability with sensor size decreasing [3,4]. Such drawbacks become critical when studying composites that are essentially graded. Use of piezoelectric low-dimensional fibers with diameters ranging from 10 to 100 µm, having a low distorting effect on the local mechanical properties of the composite matrix, seems to be a promising solution [3]. However, such a testing method requires electrical field supply plates that occupy a substantial space.

In this context, amorphous ferromagnetic materials in filament form are emerging as a highly promising solution [5]. These materials, commonly referred to as microwires, are produced through rapid quenching techniques and exhibit unique properties, such as the giant magnetoimpedance (GMI) effect [6,7] or magnetic bistability [8,9], in their amorphous state. The GMI effect refers to the significant change in electrical impedance of the material when subjected to an external magnetic field, making it highly sensitive to environmental changes such as mechanical stress, temperature, and strain [6,7]. Being easily integrated into composite structures, microwires can act as distributed sensors that respond to changes in stress or deformation, enabling new functionalities in smart structural materials.

This work aligns with this line of innovation. The main objective is to validate the potential of using composites with embedded ferromagnetic microwires as wireless stress sensors. To this end, the scattering parameters of the samples under different conditions were analyzed using a Vector Network Analyzer (VNA). This type of characterization allows for the detection of electromagnetic resonances, the shift in which under applied stress could serve as a direct indicator of the material’s structural state.

Although previous studies have evidenced a relationship between applied stress and the detected resonance frequency [10], significant variability across measurements has hindered practical implementation. This study presents a detailed analysis of the measurement system’s repeatability and the influence of factors such as the sample’s position, orientation, and mounting conditions on the results. The systematic characterization performed will establish the basis for the development of more reliable and robust sensors and contribute to the advancement of smart composite materials with structural diagnostic capabilities in real-world applications.

## 2. Materials and Methods

### 2.1. Integration of Microwires into Composite Specimens

The amorphous ferromagnetic Co_72_Fe_4_B_13_Si_11_ glass-coated microwires used in this study were produced using Taylor-Ulitovsky, as described elsewhere [11,12]. These microwires feature a 40 µm in diameter metallic nucleus, coated with a protective glass layer that stabilizes their geometry and magnetic properties. In our recent publication, we reported on a GMI ratio above 700% in such Co_72_Fe_4_B_13_Si_11_ glass-coated microwires [13].

The characteristic GMI effect of these materials depends on various parameters such as the frequency of the applied alternating current, wire geometry, alloy type, and temperature. Additionally, when mechanical stress is applied, the distribution of magnetic domains inside the microwire is altered, leading to a change in its electromagnetic response and even in stress-impedance effect [14]. This response can be detected through reflection or transmission measurements of electromagnetic waves, enabling the construction of passive, remote, and wireless sensors embedded within structural components [15,16].

To study this property, a composite laminate was fabricated, reinforced with two unidirectional fiberglass layers oriented at 0/90°, impregnated with Sicomin SR Infugreen 810 epoxy system and SD 8822 hardener in a 100:31 ratio (Sicomin, Châteauneuf les Martigues, France). Before molding, the microwires were placed equidistantly between the two reinforcement layers with a spacing of 5 mm, using a template with parallel rails. After stacking the layers and resin impregnation, a flat panel was molded in a closed vacuum press for 3 h at 120 °C, resulting in a final sample measuring 596 mm × 582 mm × 2 mm and weighing 1043.9 g.

Heating and humidity inherent in the curing process of the studied composite materials can affect the magnetic softness and the GMI effect behavior of magnetic wires [17,18,19]. Recently, we showed that heating of Co-rich microwires below 150 °C affects the GMI in a reversible way: after such heating, the GMI effect and its dependence on applied magnetic field remain almost unchanged [19]. Therefore, the irreversible changes in the GMI effect can be observed only at heating above 150 °C. Crosslinking reaction and composite molding were performed under a closed system in a vacuum bag, assuring the control of both parameters. In addition, the microwires used are covered with an insulating glass coating. This allows for increased corrosion resistance and reduced impact of humidity on the properties of the microwire used.

### 2.2. System Setup

The measurement system is based on the use of a Vector Network Analyzer, VNA, P5024B (Keysight Technologies, Inc, Santa Rosa, CA, USA) which characterizes the response of materials or devices to electromagnetic wave propagation. The parameters provided by the VNA, known as scattering or S-parameters, quantify the relationships between incident, reflected, and transmitted waves. Although all four involved S-parameters (2 in reflection and 2 in transmission) were obtained, this study primarily focused on S_22_, which measures reflection at port 2, and S_12_, which measures reverse transmission from port 2 to port 1, due to the symmetry of the sample and setup configuration.

Prior to testing, the Thru-Reflect-Match (TRM) calibration method was used, with data acquired over a frequency range of 2 GHz to 18 GHz. Two Flann DP240 dual-polarized horn antennas (Flann Microwave Ltd., Bodmin, UK) oriented along the microwire longitudinal direction were used for the measurements. These antennas allow contactless data acquisition in a free-space configuration.

For the experimental setup, the two antennas were placed facing each other at a distance of 70 cm, on either side of the SERVOSIS MUF10 tensile testing machine (Servosis S.L., Madrid, Spain) holding the sample. The sample was clamped with specially designed grips to ensure proper alignment and allow progressive application of force and stress. Signals were sent from the VNA through one antenna, passed through the sample, and recorded by the receiving antenna.

Additionally, to filter out external interferences and focus on the signal peak corresponding to the sample, a time-domain gating adjustment was performed. At each test, measurements of the S parameters were obtained at different force levels (in 1 kN increments) until the specimen was released from the jaw. To assess the repeatability of the results, these tests were repeated over five consecutive days at a rate of four tests per day.

Recently, the frequency dependence of the maximum GMI ratio of the studied microwires has been measured up to a frequency of 130 MHz [19]. The highest GMI ratio is observed at about 40 MHz, while a rather high GMI ratio is observed in the whole frequency range up to 130 MHz. On the other hand, a substantial GMI effect at GHz frequencies has been reported in Co-rich magnetic microwires with similar chemical compositions [20,21]. The advantage of the GHz frequency is the possibility of contactless GMI effect measurements, which was finally proposed for the Free Space microwave sensing technique based on tunable effective permittivity at the GHz range in composites with magnetic wire inclusions [15,16].

### 2.3. Data Processing

The signals recorded by the VNA were exported in CSV format, and the data were processed using custom code written in GNU Octave. The developed code was designed to identify resonance frequencies in the S_22_ parameter curves and analyze their shift under applied force. This allowed for the calculation of sensitivity (frequency variation per unit force) and a statistical analysis of result variability.

### 2.4. Setup Evaluation: Influence of Position and Orientation

Due to physical constraints of the experimental environment, it was not possible to work in the antennas’ far-field zone, where electromagnetic waves can be considered planar. Instead, measurements were taken in the Fresnel zone (radiated near-field), which may introduce additional errors and account for some of the observed variability. However, taking into account the planar geometry of the coupons and boundary conditions at the lab scale, this setup is representative of practical embedded system applications, where space limitations are common.

One of the main findings of the experimental phase was that small variations in sample placement (displacements or rotations) cause significant changes in the resonance frequency under tensile stress. To quantify this variation, controlled tests were conducted involving displacements along the *X* and *Z* axes, rotations around the *Y* and *Z* axes, and torsion around the *Z* axis.

## 3. Results

The measurements allowed for the analysis of the sample’s electromagnetic behavior as a function of the applied tensile force. Scattering parameters under different stress states were compared, and the influence of microwires on the material’s response was evaluated.

**Figure 1:**
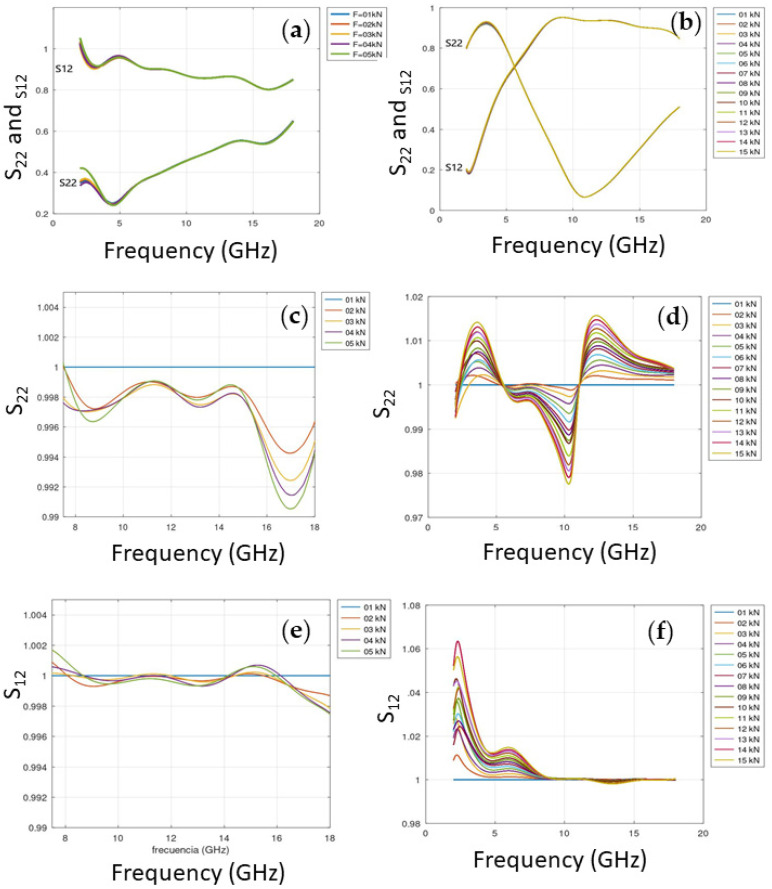
S-parameters vs. frequency (GHz) for each applied force. (**a**,**c**,**e**) S_22_ and S_12_ for the sample without microwires. (**b**,**d**,**f**) S_22_ and S_12_ for the sample with microwires.

As shown in Figure 1, the inclusion of microwires in the fiberglass matrix introduced a reflection resonance around 11 GHz, indicating a change in the material’s impedance compared to the sample without microwires. Moreover, normalizing all data by the value obtained at 1 kN enhanced the differences among them and confirmed the dependency of the S-parameter on the applied stress. This behavior does not appear in the specimen without microwires.

**Figure 2:**
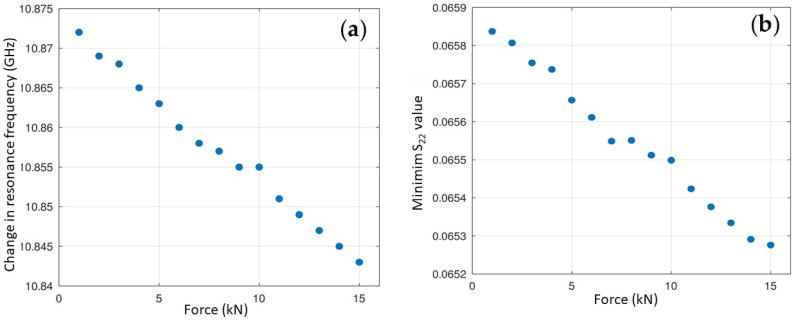
(**a**) Change in resonance frequency (GHz) and (**b**) minimum S_22_ value vs. applied force (kN).

Notably, as the tensile force increases, the resonance frequency, *F_r_*, decreases linearly (Figure 2), suggesting a direct correlation between the applied mechanical stress and the electromagnetic response.

To assess result repeatability, multiple tests were conducted on different days using the same experimental protocol. In all cases, the observed trend and the change in resonance frequency with applied load were replicated.

**Figure 3:**
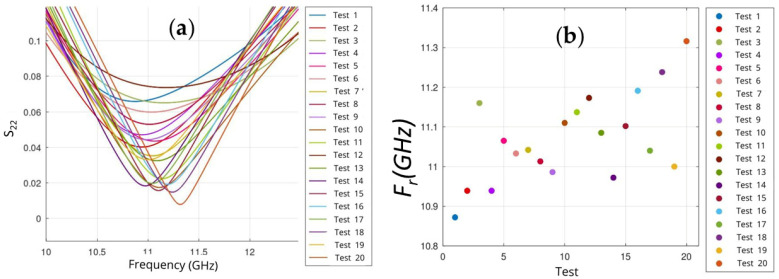
(**a**) S_22_ parameter and (**b**) resonance frequency from all tests vs. frequency (GHz) at a stress of F = 1 kN.

However, significant variability was also identified in the S-parameter values across tests (Figure 3). This uncertainty indicated that external factors were affecting the measurements, with the main one being the sample’s relative position.

**Figure 4:**
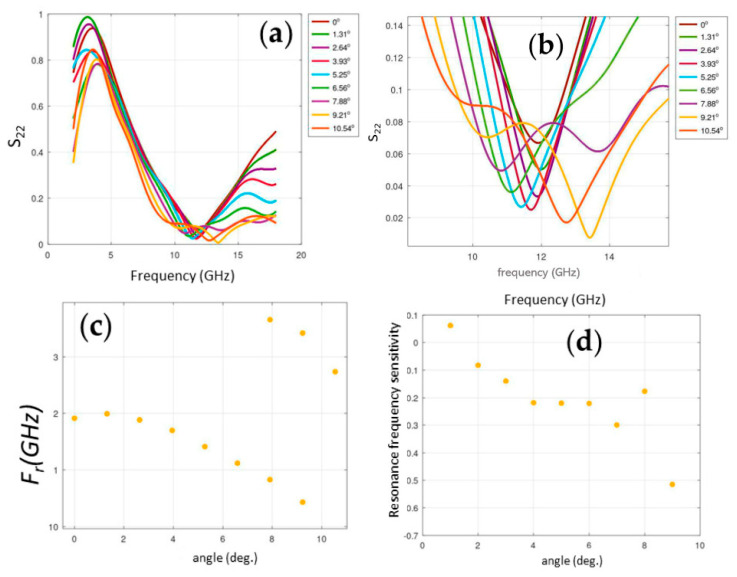
Sample response to rotation around the *Z*-axis. (**a**,**b**) S_22_ parameter vs. frequency (GHz) for the rotation angle (deg). (**c**) Resonance frequency (GHz) vs. rotation angle (deg). (**d**) Sensitivity of resonance frequency to rotation.

For this reason, the impact of sample position and orientation on result variability was studied. Displacements along the *X* and *Z* axes and rotations around the Y and Z axes were performed. It was observed that the resonance frequency is highly sensitive to these changes. As expected, displacements along the *X*-axis and rotations around the *Z*-axis caused the largest frequency variations (Figure 4), indicating that precise sample alignment is crucial for reproducible measurements.

It was calculated that the maximum sensitivity to *X*-axis displacement reaches 0.116 GHz/mm, while rotation around the *Z*-axis can generate variations up to 0.514 GHz/°. These figures far exceed the average sensitivity observed under tensile force (~0.002 GHz/kN), which implies that initial setup precision is critical to ensure system reproducibility.

This suggests that improving measurement accuracy requires the implementation of a more stable mounting system to minimize unintentional sample displacements and rotations.

## 4. Discussion

The approach followed in this study has validated the use of ferromagnetic microwires embedded in composites as wireless stress sensors. The existence of a resonance frequency associated with the presence of microwires has been demonstrated, and its shift was found to be reproducible under certain mounting conditions. In line with the previous studies and analyses, it has been observed that the S_22_ parameter and the resonance frequency decrease systematically with the increase in the applied stress on the sample. This behavior suggests that the frequency shift is due to the stress experienced by the microwires. It is worth noting that a direct correlation between the applied mechanical stress and the electromagnetic response produced by a single Co-rich microwire has been recently reported [22].

The observed effect of the applied mechanical stress on the S_22_ parameter must be related to the dependence of microwave scattering of composites with magnetic microwire inclusions on magnetic permeability of magnetic microwires and stress dependence of the GMI effect [22,23]. Particularly, it was demonstrated that the microwave scattered intensity produced by a single microwire can be affected by the magnetic permeability [24].

However, test repeatability critically depends on the accuracy of sample placement. Analyzing the contribution of each factor to overall variability showed that changes in sample position and orientation are the main contributors, exceeding the effect of the applied tensile force itself. During the study of the influence of the orientation of the sample, it has been found that the measurement system is more sensitive to changes in the spatial arrangement of the sample than to changes produced by traction. Results show that it is strongly recommended to use an automatic positioner to ensure the initial position of the coupon in order to achieve system repeatability. In order to improve the accuracy of the measurements and minimize the observed errors, several solutions are proposed. One option would be to increase the applied force to a range where the sensitivity of the measurement system overcomes the error introduced by the position and orientation of the sample. This would allow the effects of applied force to be reflected more clearly in the resonant frequency, minimizing the influence of other factors.

In addition, fixing the specimen properly would reduce those errors related to displacement. To this end, an improved design of the clamping system can be carried out, using jaws with a firmer grip to prevent slippage during tests.

The observed sensitivity of the S_22_ parameter of the composites with magnetic microwire inclusions to applied stress is suitable for wireless stress monitoring.

The proposed method allows for non-destructive wireless composites monitoring. The microwires used have excellent mechanical properties [25,26]. The use of glass-coated microwires allows for high corrosion resistance. Therefore, their use is expected to have minimal distorting effect on the local mechanical properties of the composite matrix.

Most of the traditional sensing systems for structural health monitoring (SHM) are not wireless and require data transmission from the sensor to the data processing system [27]. Such methods are expensive due to the use of expensive and long cables and associated protective pipelines [27].

Accordingly, wireless SHM systems gained great attention in recent years. Such wireless systems can potentially provide an efficient, reliable, flexible, and cost-effective scheme for SHM. However, conventional wireless sensors often remain quite expensive. That is why radio frequency identification (RFID)-based sensors were developed and proposed for SHM [28].

However, most proposed methods (regardless of technology) involve placing a chip or sensors on the surface. The use of embedded microwires allows for avoiding this limitation.

## 5. Conclusions

This study was performed on flat composite structures with integrated Co-rich ferromagnetic microwires. Flat coupons were strained in the same orientation as the microwires, and simultaneously, S parameters were determined by means of a VNA.

We have observed a clear dependence of scattering parameters and impedance on applied stress in composites with magnetic microwire inclusions. The influence of the relative sample position on these measurements has been analyzed, and the results obtained by modifying the position and orientation of microwires through rotation and flipping movements of the test specimen are presented. Observed phenomena can be useful for contactless monitoring of the composites.

## Data Availability

The data presented in this study are available on request from the corresponding author.

## Abbreviations

The following abbreviations are used in this manuscript:

GMI	Giant magnetoimpedance
VNA	Vector Network Analyser
TRM	Thru-Reflect-Match
CSV	Comma-Separated Values
